# Differential expression of microRNAs in retinal vasculopathy caused by selective Müller cell disruption

**DOI:** 10.1038/srep28993

**Published:** 2016-07-04

**Authors:** Sook Hyun Chung, Mark Gillies, Michelle Yam, Ying Wang, Weiyong Shen

**Affiliations:** 1 Macula Research Group, Clinical Ophthalmology and Eye Health, Save Sight Institute, the University of Sydney, Sydney, Australia

## Abstract

Vascular changes and photoreceptor degeneration are features of age-related macular degeneration, diabetic retinopathy and macular telangiectasis. We have profiled the differential expression of microRNAs and analysed their target genes in transgenic mice in which induced Müller cell disruption results in photoreceptor degeneration, vascular leak and deep retinal neovascularisation. We identified 9 miRNAs which were differentially expressed during the development of retinal neovascularization and chose miR-200b and its target genes for further study. Using qRT-PCR and western blot analysis, we found that downregulation of miR-200b was negatively correlated with its target genes, including zinc finger E-box binding homeobox (ZEB) 1 and 2 and vascular endothelial growth factor receptor 1. Double immunofluorescence labelling revealed that the newly formed vessels in the outer retina were positive for ZEB2. Furthermore, intravitreal injections of a miR-200b-mimic and anti-miR-200b confirmed the negative correlation of miR-200b and its target gene expression. We also found that the miR-200b-mimic inhibited vascular leak in the established mild vascular lesions, whereas anti-miR-200b promoted it. Taken together, these data suggest that miR-200b may play a role in the development of intraretinal neovascularisation.

MicroRNAs (miRNAs) are small, non-coding RNA molecules, which regulate post-transcriptional gene expression by binding to complementary sites on the 3′ untranslated region (UTR) of target genes. miRNAs have been recognized as a major player in post-transcriptional regulation of gene expression^1^. A single miRNA can modulate a wide range of genes since a short complementary sequence site to 3′UTR is required and imperfect complementary binding can still modulate target gene expression^1^,^2^.

There is increasing evidence that miRNAs play important roles in cellular proliferation, differentiation and cell death and are involved in all aspects of the biological processes investigated thus far^3^. Recent studies have reported that miRNAs play a role in the development of vasculopathy, such as endothelial migration and proliferation, and tumour angiogenesis^4^. In addition to *in vitro* and *in vivo* laboratory evidence, clinical assessment of circulating miRNAs in patients with coronary artery disease has reported high levels of pro-angiogenic miRNAs in the blood^5^. There is increasing evidence that miRNAs are also implicated in the pathogenesis of retinal degeneration, blood retinal barrier breakdown and retinal angiogenesis^6^,^7^,^8^,^9^.

Retinal vascular diseases, such as diabetic retinopathy and retinal vein occlusion, are leading causes of blindness and are often accompanied by blood retinal barrier breakdown and ocular neovascularisation^10^,^11^. Although there are now a number of treatments available for retinal vascular diseases, including intravitreal injections of steroids and antibodies against vascular endothelial growth factor, laser photocoagulation and vitrectomy, these treatments also have limitations^12^,^13^,^14^,^15^. For example, it has been well documented that overexpression of vascular endothelial growth factor (VEGF) promotes blood retinal barrier breakdown and ocular neovascularization^16^,^17^, however, recent studies suggest that long-term delivery of anti-VEGF agents may have unexpected local and systemic adverse effects^18^,^19^,^20^,^21^,^22^,^23^. Therefore, the development of other ways to treat retinal vascular diseases is warranted.

Müller cells span the retina from the internal to the external limiting membranes. They ensheath blood vessels in the plexiform and nerve fiber layers as well as all retinal neurones. Müller cells are important for the maintenance of retinal homeostasis and are involved in regulation of the blood retinal barrier, thereby controlling retinal blood supply and angiogenesis^24^. In order to study the role of primary Müller cell dysfunction in retinal diseases, we have generated transgenic mice in which induced Müller cell disruption leads BRB breakdown and deep retinal neovascularisation as well as photoreceptor degeneration^25^. These changes are important features of retinal diseases such as diabetic retinopathy, retinal vein occlusion, macular telangiectasis type 2 and some forms of age-related macular degeneration^26^,^27^,^28^,^29^.

We have recently reported the profile of differential expression of miRNAs and their target genes during the phase of photoreceptor degeneration which peaks around 2 weeks after Müller cell disruption in this model^30^, identifying 20 miRNAs and 78 target genes^31^,^32^. Since intraretinal neovascularisation appears from 2 months and persists for at least another 3 months after the induced Müller cell disruption^25^, in this study we have profiled differential expression of miRNAs and their target genes, with particular attention to the contribution of miR-200b-3p to retinal vasculopathy, 3 months after Müller cell disruption.

## Results

### miRNA profiling and target gene prediction

**Figure 1:**
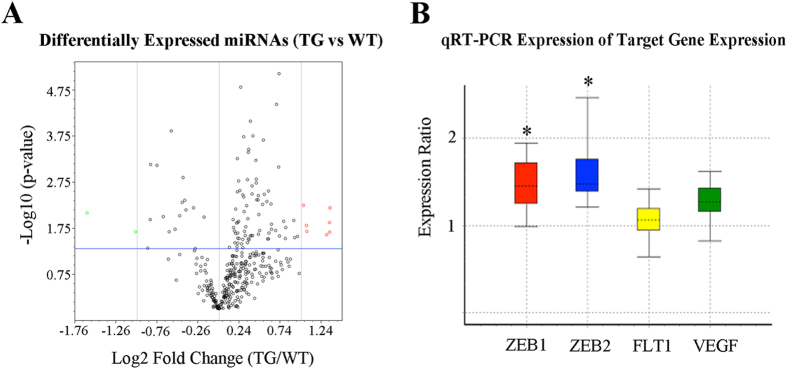
Differentially expressed miRNAs 3 months after Müller cell disruption and its target gene validation with qRT-PCR. (**A**) Volcano plot showing differentially expressed miRNAs 3 months after selective Müller cell disruption. Red circles represent upregulated miRNAs and green circles indicate downregulated miRNAs. Vertical grey lines indicate fold changes, with a cut off ±2. The horizontal blue line represents a p-value of 0.05. n = 6 in each group. (**B**) qRT-PCR analysis of genes targeted by miR-200b after selective Müller cell disruption. qRT-PCR was conducted using retinas collected 3 months after induced Müller cell disruption. The boxes represent the interquartile range. The dotted lines within the boxes represent medians of gene expression. The whiskers indicate the maximum and minimum values of gene expression. *P < 0.05, transgenic vs wild type, n = 6 in each group.

We firstly performed microRNA PCR array to profile differential expression of miRNAs 3 months after Müller cell disruption, at which time vascular leak and deep retinal neovascularisation are well established in this model. We identified 9 miRNAs, which were significantly differentially expressed. These included miR-133a-3p, miR-133b-3p, miR-146b-3p, miR-200a-3p, miR-200b-3p, miR-215-5p, miR-223-3p, miR-1936 and miR-202-3p (Fig. 1A and Table 1). Of these, miR-200 family members, including miR-200a-3p and miR-200b-3p, were significantly downregulated whereas all others were upregulated (Fig. 1A and Table 1). We next used two target gene prediction databases, TargetScan (http://www.targetscan.org) and miRTarbase (http://mirtarbase.mbc.nctu.edu.tw), to predict genes which could be potentially targeted by the 9 miRNAs. *In silico* analysis and a literature search-based functional analysis revealed which biological functions these differentially expressed miRNAs might be involved in (Table 1). Interestingly, these changes in the miR-200 family 3 months after Müller cell disruption were consistent with the findings reported by others in diabetic retinas^7^.

We next focused on miR-200b-3p since previous studies had reported that it and its target genes play an important role in angiogenesis^7^,^33^,^34^. Analysis using target gene databases revealed that there were more than 500 genes that could be targeted by miR-200b-3p. Of these, we chose 3 genes, including zinc finger E-box binding homeobox 1 and 2 (ZEB1 and ZEB2), which had the highest matching scores in analysis using conserved seed matching sequence alignments (Table 2), and vascular endothelial growth factor receptor 1 (VEGFR1, also termed FLT1), for further validation by qRT-PCR and western blot.

### Target gene validation by qRT-PCR and Western blots

We performed qRT-PCR to study changes in ZEB1, ZEB2, FLT1 and VEGF-A expression 3 months after Müller cell disruption. The primer sequences are listed in Supplementary Table 1. We found that all 4 genes were upregulated (Fig. 1B). Of these, changes in ZEB1 and ZEB2 expression were statistically significant (p < 0.05, Fig. 1B).

**Figure 2:**
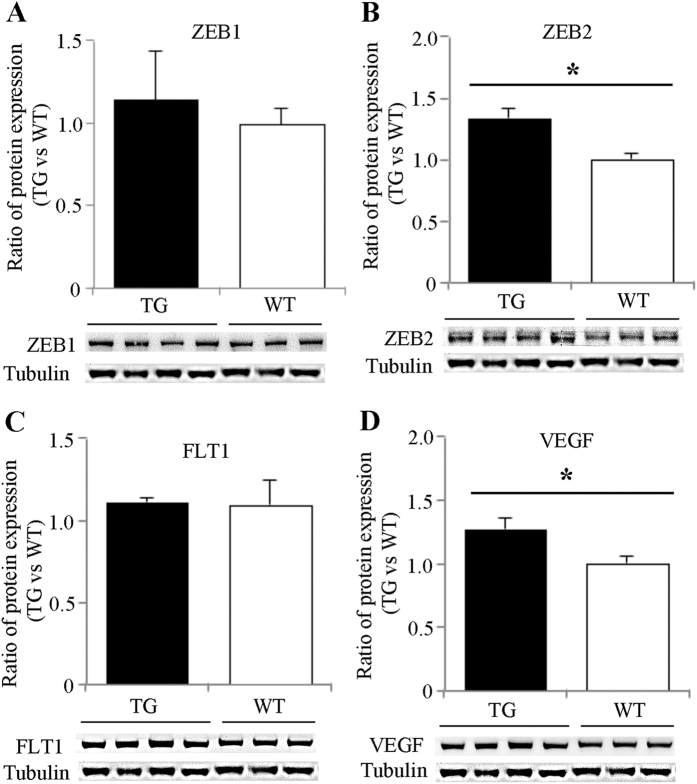
Western blot analysis of proteins targeted by miR-200b after Müller cell disruption. Western blots were performed using retinas collected 3 months after induced Müller cell disruption. *P < 0.05, transgenic (TG) vs wild type (WT), n = 6 in each group.

Western blots for ZEB1, ZEB2, FLT1 and VEGF-A revealed alterations in protein expression, which were generally consistent with the qRT-PCR. Quantitative analysis of protein bands showed significant increases in ZEB2 and VEGF-A expression, while changes in ZEB1 and FLT1 were not statistically significantly different (Fig. 2).

### Immunofluorescence labelling

**Figure 3:**
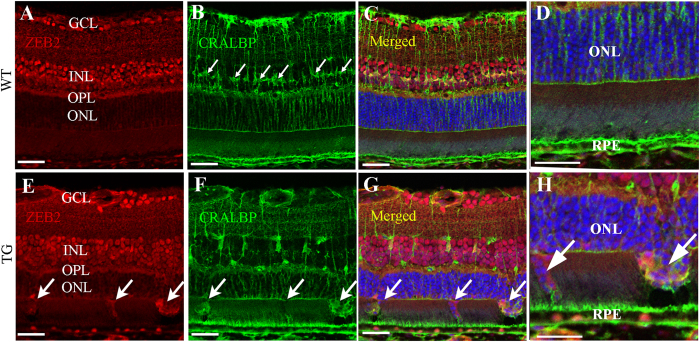
Increased immunoreactivity for ZEB2 in the outer retina after induced Müller cell disruption. An antibody against CRALBP was used to stain Müller cells. (**A**–**D**) ZEB2 was expressed in the ganglion cell layer (GCL), the inner nuclear layer (INL) and the outer plexiform layer (OPL) but hardly detected in the outer nuclear layer (ONL) in the wild type (WT) retina. The small arrows in B point to Müller cell bodies in the INL. (**E**–**H**) Transgenic (TG) retina collected 3 months after induced Müller cell disruption. Increased immunoreactivity for ZEB2 was observed in the outer retina where Müller cell ablation resulted in defects is the outer limiting membrane (small arrows in **E**–**G**) through which degenerating photoreceptor cell bodies protruded into the subretinal space (large arrows in **H**). (**D**,**H**) are higher power images of the outer retina in (**C**,**G**) respectively. RPE = retinal pigment epithelium. Scale bars: 50 μm.

We further performed immunofluorescence studies to localise the expression of ZEB2 after selective Müller cell disruption. We chose ZEB2 since it showed the most significant changes in both qRT-PCR and western blot analysis. We conducted double immunofluorescence labelling using antibodies against ZEB2 in combination with an antibody against cellular retinaldehyde binding protein (CRALBP), which is a marker for Müller cells. We found that ZEB2 was mainly expressed in the ganglion cell layer (GCL), the inner nuclear layer (INL) and the outer plexiform layer (OPL) but was hardly detected in the outer retina of the normal retina (Fig. 3A–D). Selective Müller cell disruption resulted in increased immunoreactivity for ZEB2 in some cells of the outer retina where Müller cell disruption had resulted in defects of the outer limiting membrane (Fig. 3E–G, small arrows) through which cell bodies from the outer nuclear layer (ONL) protruded into the subretinal space (Fig. 3H, large arrows). Immunostaining for ionized calcium-binding adapter molecule 1 (Iba1), a marker of microglia, indicates that the protrusion of degenerate photoreceptors (Supplementary Fig. 1G,H, large arrows) was accompanied by infiltration of activated microglia in the outer retina (Supplementary Fig. 1G,H, small arrows).

**Figure 4:**
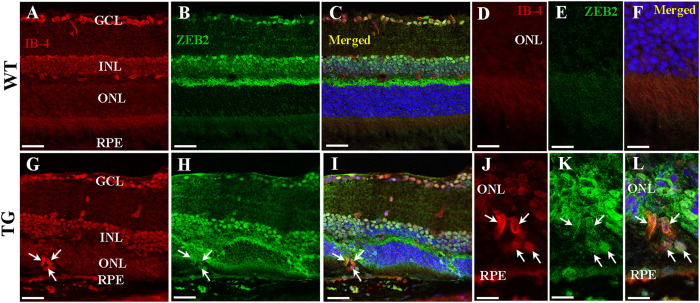
Double labelling for ZEB2 and isolectin B4 (IB-4) 3 months after Müller cell disruption. (**A**–**F**) Wild type retinas. The normal retinal vasculature is distributed within the inner retina but absent in the outer retina. (**G**–**L**) Transgenic retinas collected 3 months after Müller cell disruption. Deep intraretinal neovascularisation that had developed 3 months after induced Müller cell disruption expressed ZEB2 (arrows in (**G**–**L**). (**D**–**F**,**I**–**L**) are higher power images of (**A**–**C**,**G**–**I**) respectively. GCL-ganglion cell layer, INL = inner nuclear layer, ONL = outer nuclear layer, RPE = retinal pigment epithelium. Scale bars: 50 μm in (**A–C,G–I**); 25 μm in (**D**–**F**,**J**–**L**).

We also performed double labelling using isolectin b4 (IB-4) for blood vessels together with ZEB2 to study the relationship between abnormal vessel growth and ZEB2 overexpression (Fig. 4). The normal retinal vasculature in control mice was decorated by ZEB2 antibodies in the INL and OPL of the superficial retina (Fig. 4A–F). However, the newly developed vessels which developed in patches of Müller cell disruption showed strong immunoreactivity for ZEB2, indicating a direct association between ZEB2 overexpression and the growth of abnormal vessels in the outer retina (Fig. 4G–L).

### *In vivo* gain and loss of function studies following intravitreal injections of miR-200b regulators

In order to verify the effect of miR-200b on retinal vasculopathy *in vivo*, we performed intravitreal injections of both a miR-200b-3p mimic and inhibitor 3 months after induced Müller cell disruption. Prior to the injection, we confirmed the development of vascular lesions by fundus fluorescein angiography (FFA). The effects of the miR-200b-3p mimic and inhibitor on established vascular lesions were evaluated by re-examination with FFA 7 days after their intravitreal injection.

**Figure 5:**
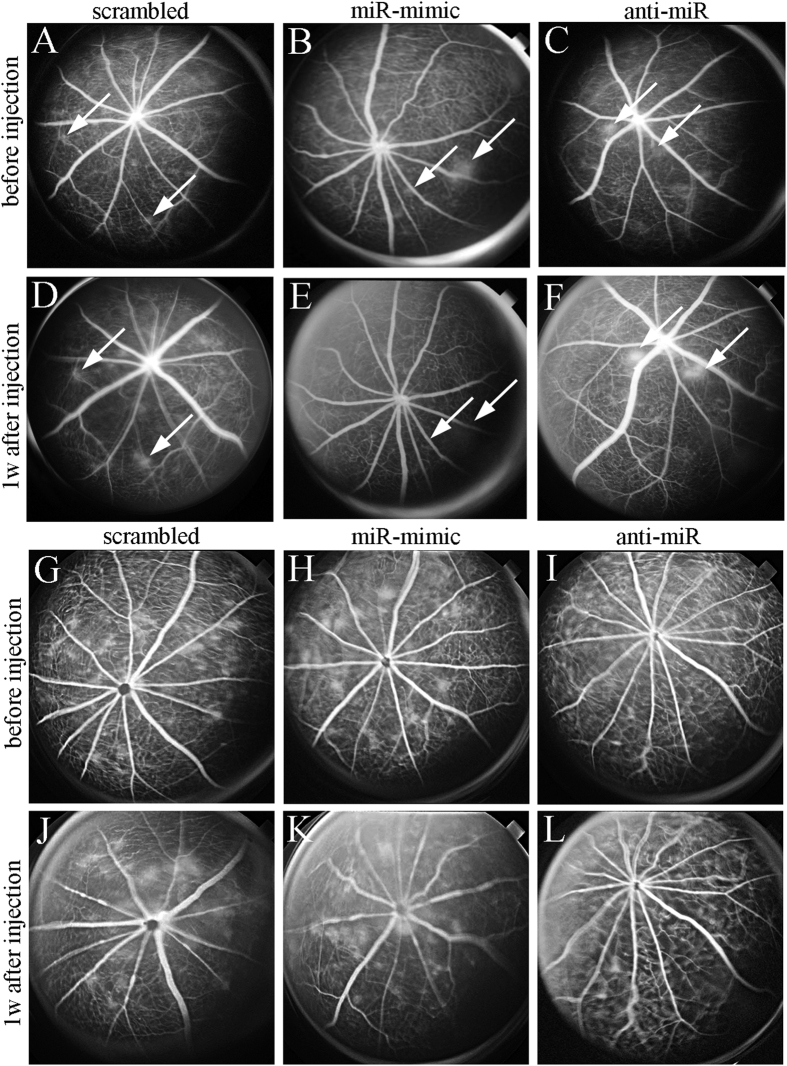
The effects of intravitreal injections of miR-200b regulators on mild (**A**–**F**) and severe (**G**–**L**) vascular lesions. (**A**–**C**,**G**–**I**) Fundus fluorescein angiograms before intravitreal injections. (**D**–**F**,**J**–**L**) Fluorescein angiograms obtained 1 week after injections. (**A**,**D**,**G**,**J**) Vascular lesions in eyes developing mild (**A**,**D**, arrows) and severe (**G**,**J**) fluorescein leak persisted before and after injection of scrambled miRNA. (**B**,**E**,**H**,**K**) miR-200b mimic inhibited vascular leak in established mild vascular lesions (arrows, **B**,**E**) but had little effects on established severe lesions (**H**,**K**). (**C**,**F**,**I**,**L**) anti-miR-200b promoted vascular leak from established mild vascular lesions (arrows, **C**,**F**) but had little effects on established severe lesions (**I**,**L**).

We analysed changes in retinae with mild and severe vascular lesions following intravitreal injections (Fig. 5). In both groups, vascular leak remained relatively unchanged 1 week after injections of a scrambled miRNA (Fig. 5A,D,G,J). Inhibition of leak from mild vascular lesions was observed in eyes receiving the miRNA mimic (Fig. 5B,E) but this effect was not obvious in eyes, which had developed severe vascular lesions before the intravitreal injection (Fig. 5H,K). As expected, anti-miR targeting miR-200b-3p appeared to promote the development of mild vascular lesions (Fig. 5C,F). However, similar to the effect of miRNA-mimic on severe vascular lesions, intravitreal injection of anti-miR showed little effect on severe vasculopathy 1 week after injection (Fig. 5I,L).

**Figure 6:**
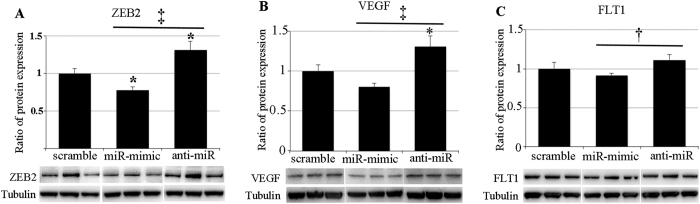
The impact of intravitreal injections of miR-200b mimic and anti-miR-200b on ZEB2, VEGF-A and FLT1 expression. Western blots for ZEB2 (**A**), VEGF-A (**B**) and FLT1 (**C**) were conducted using retinas collected 10 days after intravitreal injections of miR-200b mimic, anti-miR-200b and a scramble miRNA (control). miR-200b mimic significantly suppressed ZEB2 and tended to inhibit VEGF-A and FLT1 expression whereas anti-miR-200b significantly increased ZEB2 and VFGE-A and tended to increase FLT1 expression. The effects of miR-200b regulators on these proteins were more marked when the values of the miR-200b mimic and anti-miR-200b injected groups were compared directly. *P < 0.05, scrambled vs miR-mimic or anti-miR; ^†^P < 0.05 and ^‡^P < 0.01, miR-200b mimic vs anti-miR200b; n = 8 in miR-200b mimic group and n = 10 in anti-miR-200b group.

In order to evaluate the effects of intravitreal injections of the miR-200b-3p mimic and inhibitor on its target protein expression, we performed western blots using retinas collected from eyes receiving scrambled miRNA, the mRNA-mimic and anti-miR (Fig. 6). Consistent with the negative correlation between miR-200b-3p and its target molecules, intravitreal injection of miR-200b-3p mimic significantly suppressed or tended to decrease the expression of ZEB2 (P < 0.05), VEGF-A and FLT1, whereas anti-miR-200b-3p significantly increased or tended to stimulate the expression of these target proteins compared with eyes receiving a scramble miRNA (Fig. 6). The negative correlations between miR-200b-3p and its target proteins were more obvious when comparisons were made between mimic- and anti-miR-injected retinas (p < 0.01, Fig. 6).

## Discussion

This study identified 9 miRNAs which were differentially expressed when intraretinal neovascularisation occurred in the model we used 3 months after induced Müller cell disruption. We used multiple target gene prediction databases to predict genes which could be potentially targeted by these 9 miRNAs. We have particularly focused on the involvement of miR-200b-3p in the development of deep retinal neavascularisation through target gene validation using qRT-PCR, western blot analysis and immunofluorescence in combination with *in vivo* gain and loss of function studies after intravitreal injections of miR-200b regulators.

It has been previously reported that miRNAs are involved in ocular neovascularization in oxygen-induced retinopathy^35^,^36^ and laser-induced choroidal neovascularization^37^. This work is the first study to indicate that differentially expressed miRNAs are involved in the development of deep intraretinal neovascularization caused by prolonged primary Müller cell dysfunction.

The miR-200 family is well known for its inhibitory effect on angiogenesis through modulating transcription of various target genes such as ZEB1, ZEB2, Ets-1, VEGF, thrombospondin-1, and vasohibin-2^7^,^34^,^38^,^39^,^40^. Of this family, miR-200b is particularly important due to its involvement in the vascular complications of retinal diseases. ZEB2 has been reported to modulate tight junction and gap junction proteins, such as claudin 4, ZO-3, gap junction protein β2 and β3^41^. Our previous study using microarray analysis revealed downregulation of tight junction-associated molecules 3 months after Müller cell disruption^25^,^42^, we found significant downregulation of miR-200a-3p and miR-200b-3p 3 months after Müller cell disruption in this study. In addition, we conducted *in silico* analysis to predict which genes might have been targets of miR-200b-3p. We chose ZEB1, ZEB2 and FLT1 for further study with qRT-PCR and western blot because these molecules had the highest seed matching scores in analysis by conserved seed matching sequence alignments. Changes in VEGF-A expression were also examined due to its role in promoting vascular leak and ocular neovascularisation. qRT-PCR and western blot analysis found a negative correlation between miR-200b-3p and its target genes, particularly ZEB2. This observation is consistent with previous reports that miR-200b supresses ZEB1 and ZEB2 expression and vice versa^43^,^44^,^45^,^46^,^47^. Double immunofluorescence labelling showed overexpression of ZEB2 in abnormal vessels growing into the outer retina after selective Müller cell disruption. This is the first report that upregulation of ZEB2 may be involved in pathological ocular angiogenesis. Collectively, our data indicate that differential expression of miR-200b and its target genes may contribute to the development of vasculopathy in retinal diseases.

We conducted gain and loss of function studies to examine the effects of intravitreal injections of miR-200b mimic and anti-miR-200b in transgenic mice that had developed vascular lesions after Müller cell disruption. For the eyes which had developed mild vascular lesions, the miR-200b mimic inhibited the established vascular leak whereas anti-miR-200b promoted vascular leak. Considering the negative correlation between miR-200b and VEGF expression, future studies are warranted to test whether prolonged expression of anti-miR-200b stimulates normal blood vessels to proliferate and whether miR-200b mimic causes retinal capillary atrophy.

This study also found that neither the miR-200b mimic nor anti-miR-200b had a significant effect on established severe vascular lesions. Despite this discrepancy, data from western blots confirmed that these miRNA regulators did have the predicted effects on target gene expression. Future studies using higher doses of miR-200b mimics and anti-miR-200b are warranted to provide a better understanding of the role of miR-200b in different stages of retinal vasculopathy. It is possible that the lack of effect of miR-200b regulators on more severe vasculopathy might reflect a wide spectrum of interactions between miRNAs and their target gene expression. For example, the biological functions of one gene can be regulated by multiple miRNAs and vice versa^1^,^2^. We also acknowledge that the miRNeasy Mini Kit we employed only screens selected 372 miRNAs. Therefore, there is a possibility that we could have missed out certain miRNAs which may also play a potential role in the development of retinal vasculopathy caused by selective Müller cell disruption.

In summary, we have profiled the differential expression of miRNAs in transgenic mice in which selective Müller cell disruption results in deep intraretinal neovascularisation. Our data indicate that miR-200b and its target gene ZEB2 may play an important role in the development of intraretinal neovascularisation caused by prolonged Müller cell dysfunction.

## Materials and Methods

### Animals

This study was performed in accordance with the Association for Research in Vision and Ophthalmology guidelines for animal research and was approved by the University of Sydney animal ethics committee. Rlbp1-CreER mice were crossed with Rosa-DTA176 mice to produce Rlbp-CreER-DTA176 transgenic mice as reported previously^25^. Müller cell disruption was induced by daily intraperitoneal injection of tamoxifen for 4 consecutive days at 8–10 weeks of age^25^. Wild type mice receiving TMX in the same way were used as controls. Fundus fluorescein angiography was performed periodically to monitor retinal vascular changes after Müller cell disruption as described previously^25^. Mice were euthanized 3 months after Müller cell disruption. Retinae were isolated, snap frozen in liquid nitrogen and stored at −80 °C until use. In addition, eyes were also fixed with 4% paraformaldehyde and embedded in Optimal Cutting Temperature compound.

### MicroRNA profiling and target gene prediction

A miRNeasy Mini Kit (Qiagen, 217004) was used to extract miRNAs according to the manufacturer’s instructions. 1 μg of total RNA from each of 12 retinae (6 transgenic, 6 wild type) were reverse-transcribed with a miRNA specific reverse transcript kit, miScript II RT (Qiagen, 218160), and miRNA PCR array was performed with 384 well-plate miScript miRNA High Content PCR array (Qiagen, MIMM-3001Z) which contains miScript primers for 372 of the most well characterised miRNAs. The identities of 372 miRNAs are listed in Supplementary Table 2. The thermo-cycle of the PCR array were denaturing at 94 °C for 15 seconds, annealing at 55 °C for 20 seconds and extension at 70 °C for 30 seconds with a total of 40 cycles. Relative quantification was performed by the ∆∆C_T_ method as recommended by the manufacturer (http://pcrdataanalysis.sabiosciences.com/mirna/arrayanalysis.php?target=analysis). A p-value < 0.05 was considered statistically significant and the fold change cut-off criterion was ±2.

### Quantitative RT-PCR (qRT-PCR)

qRT-PCR was performed to validate target gene expression as previously described^42^. In brief, total RNA was extracted and cDNA synthesis was performed with a SuperScript VILO cDNA Synthesis kit (Life Technologies, 11754050). Diluted cDNA was used for PCR reaction with Express SYBR GreenER qPCR Supermix (Life Technologies, 11784-200) according to manufacturer’s instruction. The quantitative analysis was performed by Relative Expression Software Tool (REST) 2009^48^. Expression values were normalised with 2 reference genes including 18SrRNA and GAPDH.

### Western blot analysis

Western blot was performed as previously described^42^. Briefly, retinae were thawed and protein extraction was performed with RIPA buffer (Sigma, R0278) containing protease inhibitor (Roche, 04693159001), and QuantiPro BCA assay kit (Sigma-Aldrich, QPBCA) was used for protein quantification. Equal amounts of protein were loaded into each lane of NuPage Bis-Tris gels (Life Technologies, NP3023BOX), and transferred to a polyvinylidene difluoride membrane with semi-dry transfer system. The membranes were blocked with 5% BSA in TBST and the primary antibodies were incubated overnight at 4 °C. Primary antibodies include ZEB1 (Santa Cruz, sc-25388), ZEB2 (Abcam, ab25837), FLT1 (Abcam, ab32152), and VEGF (Abcam, ab1316). The horseradish peroxidase secondary antibody incubation was followed for 2–4 hours at room temperature. After ECL incubation, protein bands were visualised with GeneTool image scanning and densitometry measurement. The results were normalised with loading control of α/β tubulin (Cell Signalling, #2148).

### *In vivo* gain and loss of function studies following intravitreal injections of miR-200b regulators

We performed intravitreal injections of miR-200b mimic (mirVana mimics, life technologies), miR-200b inhibitor (mirVana inhibitors, life technologies) to study the effects of miR-200b regulators on retinal vasculopathy 3 month after induced Müller cell disruption. A scrambled miRNA (mirVana negative control, life technologies) was injected in the same way as a control. A total of 18 mice were used for *in vivo* study: 8 mice received miR-200b mimic in one eye and the contralateral eye in each mouse received scrambled miRNA for comparison, with 2.8 μg/eye. Similarly, anti-miR-200b and scrambled RNA were injected in 10 mice. We used MaxSuppressor^TM^ *in vivo* RNA-LANCErII (Bioo Scientific) as a miRNA mediator for efficient miRNA delivery. One week before intravitreal injection, we performed FFA to confirm the development of retinal vascular lesions after selective Müller cell disruption. The injected animals were re-examined for FFA to monitor changes in vascular lesions after intravitreal injections.

### Immunofluorescence labelling

Immunofluorescence labelling was performed as previously described^42^,^49^. Briefly, eye cups were fixed with 4% paraformaldehyde in PBS for an hour at room temperature and incubated with 20% sucrose in PBS overnight at 4 °C. Then the eyecups were embedded in OCT, sectioned at 12 μm on Superfrost glass slides and stored at −20 °C until use. For fluorescent immunostaining, sections were thawed and washed with PBS 3 times for 5 minutes and blocked with 10% normal goat serum in PBS for 1 hour at room temperature. After washing with PBS, primary antibody incubation was followed for overnight at 4 °C. The primary antibodies used in this study were ZEB1 (Santa Cruz, sc-25388), ZEB2 (Abcam, ab25837), and cellular retinaldehyde binding protein (CRALBP, Abcam, ab15051). Isolectin B4 (IB4, Invitrogen I21413) was used to label blood vessels as previously described^25^. Secondary antibody incubation was performed the next day for 2–4 hours at room temperature followed by nuclear counterstaining with Hoechst. Sections were examined by confocal microscopy as described previously^25^,^42^.

### Statistical analysis

Results were expressed as mean ± SEM. Data were analysed using unpaired Student t-test. A p value < 0.05 was regarded as statistically significant.

## Additional Information

**How to cite this article**: Chung, S. H. *et al*. Differential expression of microRNAs in retinal vasculopathy caused by selective Müller cell disruption. *Sci. Rep.* **6**, 28993; doi: 10.1038/srep28993 (2016).

## Supplementary Material

Supplementary Information

## Figures and Tables

**Table 1:** List of differentially expressed miRNAs 3 months after Müller cell disruption.

miRNA ID	P-value	Fold Change	Functional analysis based on literature research	Potential Target Genes	References
mmu-miR-133a-3p	0.02	2.46	Cell proliferation and integration, Energy metabolism	ERK1/2 Prdm16	50,51
mmu-miR-133b-3p	0.02	2.55			
mmu-miR-146b-3p	0.02	2.10	Energy Metabolism, Cell Differentiation, Innate Immunity	Sirt1 IRAK1 TRAF6	52,53
mmu-miR-200a-3p	0.008	−3.05	VEGF signaling, Angiogenesis	VEGF VASH2 THBS1 FLT1 KDR	7,33,34,38,39,54,55
mmu-miR-200b-3p	0.02	−2.03			
mmu-miR-202-3p	0.015	2.10	Apoptosis, sexual differentiation	GLI1, GLI2, SOX9	56, 57, 58
mmu-miR-215-5p	0.01	2.53	p53 pathway, Cadherin Regulation	Ecadherins P53	59,60
mmu-miR-223-3p	0.005	2.04	Neuroprotection, Energy Metabolism	NR2B GLUR2 GLUT4	61,62
mmu-miR-1936	0.006	2.55	Not well characterized		N/A

miR-200a-3p and miR-200b-3p were significantly downregulated. A literature-based functional analysis indicated that both were related to VEGF signalling and angiogenesis.

**Table 2:**
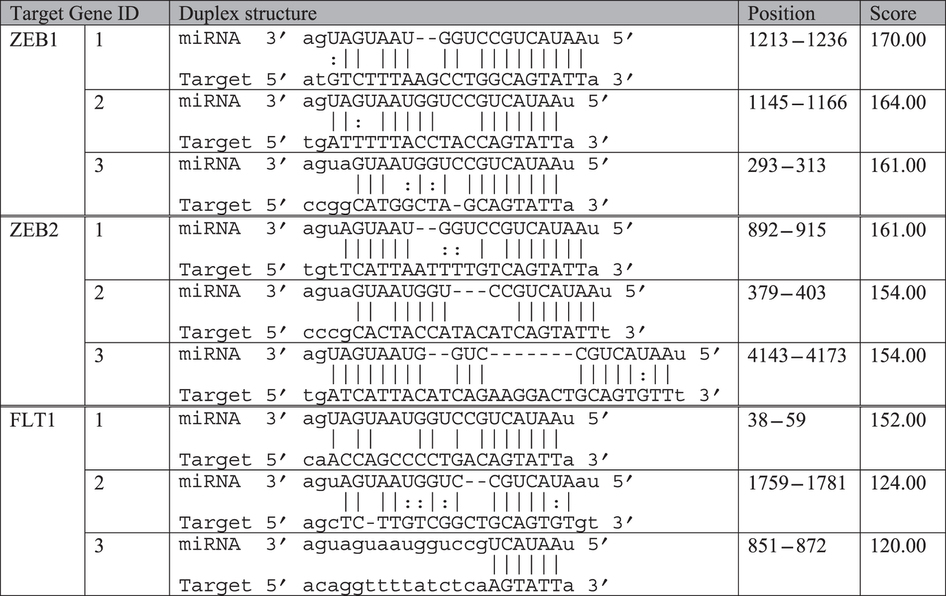
Seed matching sequence alignments between miR-200b and its target genes including ZEB1, ZEB2 and FLT1.

Analysis was conducted using two target gene prediction databases including TargetScan (http://www.targetscan.org) and miRTarbase (http://mirtarbase.mbc.nctu.edu.tw). ZEB1 and ZEB2 had highly conserved seed matching sequences as evidenced by more than 8mer sequence matching with miR-200b at all 3 positions.
