# Supplementary material for: Differential expression of microRNAs in retinal vasculopathy caused by selective Müller cell disruption
Source: Sci Rep. 2016 Jul 4;6:28993. doi: 10.1038/srep28993 (PMC4931578; doi:10.1038/srep28993)
Supplement: Supplementary Information [file srep28993-s1.doc]

***Supporting Information***

**Differential expression of microRNAs in retinal vasculopathy caused by selective Müller cell disruption**

SOOK HYUN CHUNG^1*^, MARK GILLIES^1^, MICHELLE YAM^1^, YING WANG^1^ and WEIYONG SHEN^1*^

^1^Macula Research Group, Clinical Ophthalmology and Eye Health, Save Sight Institute, the University of Sydney, Sydney, Australia


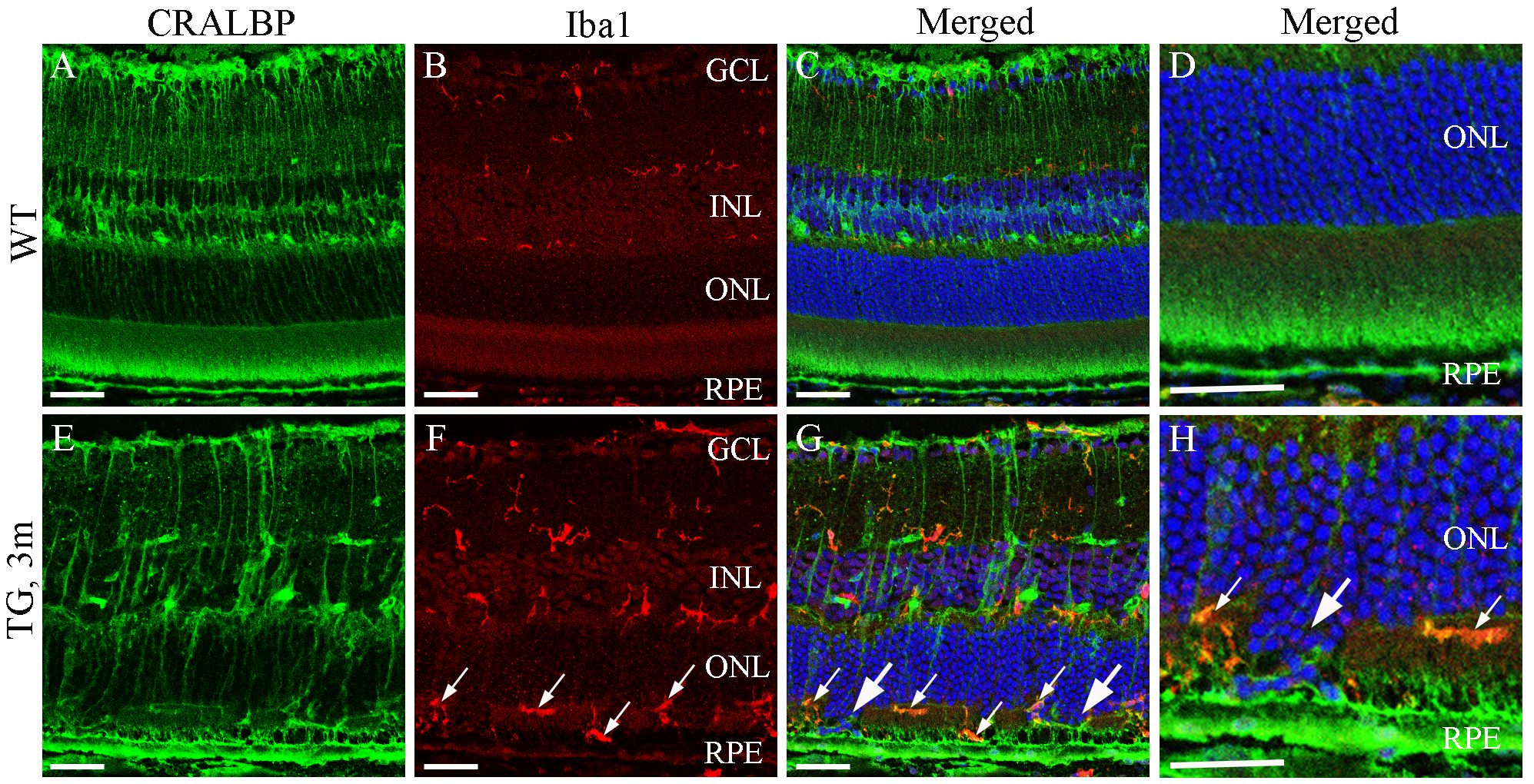


**Supplementary Figure 1. Activation of microglia after induced Muller cell disruption.** Immunostaining was conducted using antibodies to cellular retinaldehyde binding protein (CRALBP, green, Abcam, 1:200) and ionized calcium-binding adapter molecule 1 (Iba1, red, Wako, 1:500) in wild type (WT, A-D) and transgenic (TG, E-H) mice 3 months after induced Müller cell disruption. Resting microglia were confined to the inner retina in WT mice (B). Induced Müller cell disruption caused protrusion of photoreceptor cell bodies from the outer nuclear layer into the subretinal space (large arrows in G and H) and activated microglia (small arrows in F-H). Note: the cell bodies protruding from the ONL into the subretinal space are negative for Iba1, indicating that they are photoreceptors rather than inflammatory cells. (D) and (H) are high power images of an area in (C) and (G) respectively. GCL-ganglion cell layer, INL=inner nuclear layer, ONL=outer nuclear layer, RPE=retinal pigment epithelium. Scale bars: 50μm.

[Table 1] Information on primers used for qRT-PCR analysis to validate the genes targeted by miR-200b.

| **Primers** | **Forward (5’->3’)** | **Reverse (5’->3’)** |
| --- | --- | --- |
| *ZEB1* | GCTGGCAAGACAACGTGAAAG | GCCTCAGGATAAATGACGGC |
| *ZEB2* | ATTGCACATCAGACTTTGAGGAA | ATAATGGCCGTGTCGCTTCG |
| *FLT1* | CCACCTCTCTATCCGCTGG | ACCAATGTGCTAACCGTCTTATT |
| *VEGF* | GCACATAGAGAGAATGAGCTTCC | CTCCGCTCTGAACAAGGCT |
| *18srRNA* | GCAATTATTCCCCATGAACG | GGGACTTAATCAACGCAAGC |
| *Gapdh* | AAGATGGTGATGGGCTTCCCG | TGGCAAAGTGGAGATTGTTGCC |

[Table 2] The list of 372 miRNA IDs used in Qiagen HC PCR array.

| **Plate Position** | **miRNA ID** |
| --- | --- |
| A01 | mmu-let-7a-5p |
| A02 | mmu-let-7b-5p |
| A03 | mmu-let-7c-5p |
| A04 | mmu-let-7d-5p |
| A05 | mmu-let-7d-3p |
| A06 | mmu-let-7e-5p |
| A07 | mmu-let-7f-5p |
| A08 | mmu-let-7g-5p |
| A09 | mmu-let-7i-5p |
| A10 | mmu-miR-100-5p |
| A11 | mmu-miR-101a-3p |
| A12 | mmu-miR-101a-5p |
| A13 | mmu-miR-101b-3p |
| A14 | mmu-miR-103-3p |
| A15 | mmu-miR-106a-5p |
| A16 | mmu-miR-106b-5p |
| A17 | mmu-miR-107-3p |
| A18 | mmu-miR-10a-5p |
| A19 | mmu-miR-10b-5p |
| A20 | mmu-miR-122-5p |
| A21 | mmu-miR-124-3p |
| A22 | mmu-miR-124-5p |
| A23 | mmu-miR-125a-5p |
| A24 | mmu-miR-125b-5p |
| B01 | mmu-miR-126a-3p |
| B02 | mmu-miR-126a-5p |
| B03 | mmu-miR-128-3p |
| B04 | mmu-miR-129-5p |
| B05 | mmu-miR-130a-3p |
| B06 | mmu-miR-130b-3p |
| B07 | mmu-miR-132-3p |
| B08 | mmu-miR-133a-3p |
| B09 | mmu-miR-133a-5p |
| B10 | mmu-miR-133b-3p |
| B11 | mmu-miR-134-5p |
| B12 | mmu-miR-135a-5p |
| B13 | mmu-miR-135b-5p |
| B14 | mmu-miR-137-3p |
| B15 | mmu-miR-138-5p |
| B16 | mmu-miR-139-5p |
| B17 | mmu-miR-140-5p |
| B18 | mmu-miR-141-3p |
| B19 | mmu-miR-142-3p |
| B20 | mmu-miR-142-5p |
| B21 | mmu-miR-143-3p |
| B22 | mmu-miR-143-5p |
| B23 | mmu-miR-144-3p |
| B24 | mmu-miR-145a-5p |
| C01 | mmu-miR-145a-3p |
| C02 | mmu-miR-146a-5p |
| C03 | mmu-miR-146b-5p |
| C04 | mmu-miR-146b-3p |
| C05 | mmu-miR-147-3p |
| C06 | mmu-miR-148a-3p |
| C07 | mmu-miR-148b-3p |
| C08 | mmu-miR-149-5p |
| C09 | mmu-miR-150-5p |
| C10 | mmu-miR-151-3p |
| C11 | mmu-miR-151-5p |
| C12 | mmu-miR-152-3p |
| C13 | mmu-miR-153-3p |
| C14 | mmu-miR-155-5p |
| C15 | mmu-miR-155-3p |
| C16 | mmu-miR-15a-5p |
| C17 | mmu-miR-15b-5p |
| C18 | mmu-miR-16-5p |
| C19 | mmu-miR-17-5p |
| C20 | mmu-miR-181a-5p |
| C21 | mmu-miR-181b-5p |
| C22 | mmu-miR-181c-5p |
| C23 | mmu-miR-181d-5p |
| C24 | mmu-miR-182-5p |
| D01 | mmu-miR-182-3p |
| D02 | mmu-miR-183-5p |
| D03 | mmu-miR-183-3p |
| D04 | mmu-miR-184-3p |
| D05 | mmu-miR-185-5p |
| D06 | mmu-miR-186-5p |
| D07 | mmu-miR-187-3p |
| D08 | mmu-miR-18a-5p |
| D09 | mmu-miR-190a-5p |
| D10 | mmu-miR-191-5p |
| D11 | mmu-miR-192-5p |
| D12 | mmu-miR-192-3p |
| D13 | mmu-miR-193a-3p |
| D14 | mmu-miR-193b-3p |
| D15 | mmu-miR-194-5p |
| D16 | mmu-miR-195a-5p |
| D17 | mmu-miR-196a-5p |
| D18 | mmu-miR-196b-5p |
| D19 | mmu-miR-196b-3p |
| D20 | mmu-miR-199a-3p |
| D21 | mmu-miR-199a-5p |
| D22 | mmu-miR-199b-5p |
| D23 | mmu-miR-19a-3p |
| D24 | mmu-miR-19b-3p |
| E01 | mmu-miR-1a-3p |
| E02 | mmu-miR-200a-3p |
| E03 | mmu-miR-200b-3p |
| E04 | mmu-miR-200c-3p |
| E05 | mmu-miR-203-3p |
| E06 | mmu-miR-204-5p |
| E07 | mmu-miR-204-3p |
| E08 | mmu-miR-205-5p |
| E09 | mmu-miR-206-3p |
| E10 | mmu-miR-206-5p |
| E11 | mmu-miR-207 |
| E12 | mmu-miR-20a-5p |
| E13 | mmu-miR-20b-5p |
| E14 | mmu-miR-21a-5p |
| E15 | mmu-miR-21a-3p |
| E16 | mmu-miR-210-3p |
| E17 | mmu-miR-211-5p |
| E18 | mmu-miR-214-3p |
| E19 | mmu-miR-215-5p |
| E20 | mmu-miR-216a-5p |
| E21 | mmu-miR-217-5p |
| E22 | mmu-miR-217-3p |
| E23 | mmu-miR-218-5p |
| E24 | mmu-miR-22-3p |
| F01 | mmu-miR-22-5p |
| F02 | mmu-miR-221-3p |
| F03 | mmu-miR-221-5p |
| F04 | mmu-miR-222-3p |
| F05 | mmu-miR-222-5p |
| F06 | mmu-miR-223-3p |
| F07 | mmu-miR-224-5p |
| F08 | mmu-miR-23a-3p |
| F09 | mmu-miR-23b-3p |
| F10 | mmu-miR-23b-5p |
| F11 | mmu-miR-24-3p |
| F12 | mmu-miR-25-3p |
| F13 | mmu-miR-26a-5p |
| F14 | mmu-miR-26b-5p |
| F15 | mmu-miR-27a-3p |
| F16 | mmu-miR-27b-3p |
| F17 | mmu-miR-27b-5p |
| F18 | mmu-miR-28a-5p |
| F19 | mmu-miR-292-3p |
| F20 | mmu-miR-295-3p |
| F21 | mmu-miR-298-5p |
| F22 | mmu-miR-29a-3p |
| F23 | mmu-miR-29b-3p |
| F24 | mmu-miR-29c-3p |
| G01 | mmu-miR-301a-3p |
| G02 | mmu-miR-302a-5p |
| G03 | mmu-miR-302d-3p |
| G04 | mmu-miR-30a-5p |
| G05 | mmu-miR-30b-5p |
| G06 | mmu-miR-30b-3p |
| G07 | mmu-miR-30c-5p |
| G08 | mmu-miR-30d-5p |
| G09 | mmu-miR-30e-5p |
| G10 | mmu-miR-31-5p |
| G11 | mmu-miR-31-3p |
| G12 | mmu-miR-32-5p |
| G13 | mmu-miR-320-3p |
| G14 | mmu-miR-322-5p |
| G15 | mmu-miR-324-5p |
| G16 | mmu-miR-326-3p |
| G17 | mmu-miR-328-3p |
| G18 | mmu-miR-33-5p |
| G19 | mmu-miR-330-5p |
| G20 | mmu-miR-335-5p |
| G21 | mmu-miR-338-3p |
| G22 | mmu-miR-339-5p |
| G23 | mmu-miR-342-3p |
| G24 | mmu-miR-345-5p |
| H01 | mmu-miR-346-5p |
| H02 | mmu-miR-34a-5p |
| H03 | mmu-miR-34c-5p |
| H04 | mmu-miR-34c-3p |
| H05 | mmu-miR-375-3p |
| H06 | mmu-miR-376b-3p |
| H07 | mmu-miR-376c-3p |
| H08 | mmu-miR-378a-3p |
| H09 | mmu-miR-378a-5p |
| H10 | mmu-miR-381-3p |
| H11 | mmu-miR-383-5p |
| H12 | mmu-miR-409-3p |
| H13 | mmu-miR-423-5p |
| H14 | mmu-miR-425-5p |
| H15 | mmu-miR-429-3p |
| H16 | mmu-miR-431-5p |
| H17 | mmu-miR-433-3p |
| H18 | mmu-miR-451a |
| H19 | mmu-miR-467e-5p |
| H20 | mmu-miR-484 |
| H21 | mmu-miR-485-5p |
| H22 | mmu-miR-493-3p |
| H23 | mmu-miR-495-3p |
| H24 | mmu-miR-497-5p |
| I01 | mmu-miR-499-5p |
| I02 | mmu-miR-503-5p |
| I03 | mmu-miR-503-3p |
| I04 | mmu-miR-541-5p |
| I05 | mmu-miR-574-3p |
| I06 | mmu-miR-598-3p |
| I07 | mmu-miR-652-3p |
| I08 | mmu-miR-744-5p |
| I09 | mmu-miR-7a-5p |
| I10 | mmu-miR-872-5p |
| I11 | mmu-miR-880-3p |
| I12 | mmu-miR-9-5p |
| I13 | mmu-miR-92a-3p |
| I14 | mmu-miR-93-5p |
| I15 | mmu-miR-96-5p |
| I16 | mmu-miR-98-5p |
| I17 | mmu-miR-99a-5p |
| I18 | mmu-miR-127-3p |
| I19 | mmu-miR-296-3p |
| I20 | mmu-miR-30c-1-3p |
| I21 | mmu-miR-34b-3p |
| I22 | mmu-miR-367-3p |
| I23 | mmu-miR-677-3p |
| I24 | mmu-miR-709 |
| J01 | mmu-miR-714 |
| J02 | mmu-miR-762 |
| J03 | mmu-miR-1b-5p |
| J04 | mmu-miR-212-3p |
| J05 | mmu-miR-219a-1-3p |
| J06 | mmu-miR-219a-5p |
| J07 | mmu-miR-290a-3p |
| J08 | mmu-miR-335-3p |
| J09 | mmu-miR-338-5p |
| J10 | mmu-miR-377-3p |
| J11 | mmu-miR-486-5p |
| J12 | mmu-miR-1187 |
| J13 | mmu-miR-1191 |
| J14 | mmu-miR-1192 |
| J15 | mmu-miR-1195 |
| J16 | mmu-miR-1196-5p |
| J17 | mmu-miR-1224-5p |
| J18 | mmu-miR-1249-3p |
| J19 | mmu-miR-1298-5p |
| J20 | mmu-miR-135a-1-3p |
| J21 | mmu-miR-136-5p |
| J22 | mmu-miR-154-5p |
| J23 | mmu-miR-16-1-3p |
| J24 | mmu-miR-188-3p |
| K01 | mmu-miR-188-5p |
| K02 | mmu-miR-1895 |
| K03 | mmu-miR-1900 |
| K04 | mmu-miR-1901 |
| K05 | mmu-miR-1902 |
| K06 | mmu-miR-1904 |
| K07 | mmu-miR-1931 |
| K08 | mmu-miR-1934-5p |
| K09 | mmu-miR-1936 |
| K10 | mmu-miR-1940 |
| K11 | mmu-miR-1945 |
| K12 | mmu-miR-1953 |
| K13 | mmu-miR-1957a |
| K14 | mmu-miR-1963 |
| K15 | mmu-miR-1965 |
| K16 | mmu-miR-1968-3p |
| K17 | mmu-miR-1971 |
| K18 | mmu-miR-1983 |
| K19 | mmu-miR-19b-1-5p |
| K20 | mmu-miR-1a-1-5p |
| K21 | mmu-miR-1a-2-5p |
| K22 | mmu-miR-202-3p |
| K23 | mmu-miR-208a-3p |
| K24 | mmu-miR-208a-5p |
| L01 | mmu-miR-208b-3p |
| L02 | mmu-miR-2137 |
| L03 | mmu-miR-2183 |
| L04 | mmu-miR-24-2-5p |
| L05 | mmu-miR-291b-3p |
| L06 | mmu-miR-293-5p |
| L07 | mmu-miR-294-3p |
| L08 | mmu-miR-302a-3p |
| L09 | mmu-miR-302b-3p |
| L10 | mmu-miR-3099-3p |
| L11 | mmu-miR-30c-2-3p |
| L12 | mmu-miR-331-3p |
| L13 | mmu-miR-341-3p |
| L14 | mmu-miR-343 |
| L15 | mmu-miR-344d-3-5p |
| L16 | mmu-miR-350-3p |
| L17 | mmu-miR-351-3p |
| L18 | mmu-miR-361-5p |
| L19 | mmu-miR-363-3p |
| L20 | mmu-miR-363-5p |
| L21 | mmu-miR-365-3p |
| L22 | mmu-miR-374b-5p |
| L23 | mmu-miR-376a-3p |
| L24 | mmu-miR-379-5p |
| M01 | mmu-miR-382-5p |
| M02 | mmu-miR-410-3p |
| M03 | mmu-miR-421-3p |
| M04 | mmu-miR-423-3p |
| M05 | mmu-miR-434-3p |
| M06 | mmu-miR-434-5p |
| M07 | mmu-miR-448-3p |
| M08 | mmu-miR-448-5p |
| M09 | mmu-miR-449a-5p |
| M10 | mmu-miR-449b |
| M11 | mmu-miR-449c-5p |
| M12 | mmu-miR-450a-5p |
| M13 | mmu-miR-452-3p |
| M14 | mmu-miR-452-5p |
| M15 | mmu-miR-455-3p |
| M16 | mmu-miR-466d-3p |
| M17 | mmu-miR-466h-5p |
| M18 | mmu-miR-468-3p |
| M19 | mmu-miR-470-5p |
| M20 | mmu-miR-483-5p |
| M21 | mmu-miR-483-3p |
| M22 | mmu-miR-490-3p |
| M23 | mmu-miR-490-5p |
| M24 | mmu-miR-491-5p |
| N01 | mmu-miR-496a-3p |
| N02 | mmu-miR-500-3p |
| N03 | mmu-miR-501-3p |
| N04 | mmu-miR-501-5p |
| N05 | mmu-miR-504-5p |
| N06 | mmu-miR-505-3p |
| N07 | mmu-miR-532-5p |
| N08 | mmu-miR-539-3p |
| N09 | mmu-miR-539-5p |
| N10 | mmu-miR-540-3p |
| N11 | mmu-miR-540-5p |
| N12 | mmu-miR-542-5p |
| N13 | mmu-miR-546 |
| N14 | mmu-miR-568 |
| N15 | mmu-miR-582-5p |
| N16 | mmu-miR-592-3p |
| N17 | mmu-miR-653-5p |
| N18 | mmu-miR-664-3p |
| N19 | mmu-miR-667-3p |
| N20 | mmu-miR-669a-5p |
| N21 | mmu-miR-669c-5p |
| N22 | mmu-miR-670-5p |
| N23 | mmu-miR-673-3p |
| N24 | mmu-miR-674-5p |
| O01 | mmu-miR-675-3p |
| O02 | mmu-miR-675-5p |
| O03 | mmu-miR-676-3p |
| O04 | mmu-miR-679-5p |
| O05 | mmu-miR-681 |
| O06 | mmu-miR-682 |
| O07 | mmu-miR-691 |
| O08 | mmu-miR-694 |
| O09 | mmu-miR-695 |
| O10 | mmu-miR-696 |
| O11 | mmu-miR-697 |
| O12 | mmu-miR-698-3p |
| O13 | mmu-miR-701-5p |
| O14 | mmu-miR-704 |
| O15 | mmu-miR-708-5p |
| O16 | mmu-miR-710 |
| O17 | mmu-miR-711 |
| O18 | mmu-miR-712-5p |
| O19 | mmu-miR-713 |
| O20 | mmu-miR-717 |
| O21 | mmu-miR-719 |
| O22 | mmu-miR-720 |
| O23 | mmu-miR-721 |
| O24 | mmu-miR-758-3p |
| P01 | mmu-miR-761 |
| P02 | mmu-miR-763 |
| P03 | mmu-miR-764-3p |
| P04 | mmu-miR-764-5p |
| P05 | mmu-miR-802-5p |
| P06 | mmu-miR-804 |
| P07 | mmu-miR-871-3p |
| P08 | mmu-miR-871-5p |
| P09 | mmu-miR-875-3p |
| P10 | mmu-miR-877-5p |
| P11 | mmu-miR-879-5p |
| P12 | mmu-miR-883b-5p |
| P13 | cel-miR-39-3p |
| P14 | cel-miR-39-3p |
| P15 | SNORD61 |
| P16 | SNORD68 |
| P17 | SNORD72 |
| P18 | SNORD95 |
| P19 | SNORD96A |
| P20 | RNU6-2 |
| P21 | miRTC |
| P22 | miRTC |
| P23 | PPC |
| P24 | PPC |
